# Trends and outcomes of *Mycoplasma pneumoniae* pneumonia in hospitalized children before, during, and after the coronavirus disease 2019 pandemic: a retrospective study in Northeast China, 2018–2023

**DOI:** 10.1128/spectrum.00354-26

**Published:** 2026-08-14

**Authors:** Chunxue Jiang, Xin Chen, Xuewen Xu, Tingting Sun, Wei Xu, Kai You

**Affiliations:** 1 Department of Pediatrics, Shengjing Hospital of China Medical University 85024, Shenyang, China; 2 Department of Urology, Shengjing Hospital of China Medical University 85024, Shenyang, China; Ann & Robert H. Lurie Children's Hospital of Chicago, Chicago, Illinois, USA; University of Louisville, Louisville, Kentucky, USA; The University of Texas Health Science Center at San Antonio, San Antonio, Texas, USA

**Keywords:** *Mycoplasma pneumoniae* pneumonia, pediatric, COVID-19, epidemiology, public health, hospitalization

## Abstract

*Mycoplasma pneumoniae* pneumonia (MPP) is the most common form of community-acquired pneumonia, which shows an epidemic interval of 3–7 years. This study was conducted to understand the changing trends in MPP in children hospitalized before and during the coronavirus disease 2019 pandemic and determine adverse outcomes and their influencing factors. This was a retrospective cohort study of children aged 28 days to 14 years hospitalized for MPP at our institution between January 2018 and December 2023. Clinical characteristics, adverse outcomes, and associated factors were analyzed using the chi-square test and multivariable logistic regression. A *P* < 0.05 was considered statistically significant. A total of 17,505 MPP cases were included, with the highest case number (4,619) in 2023. In 2023, the proportion of severe pneumonia was 44.1%, and bronchoscopy usage was 33.5%, both significantly higher than in 2018–2022. Older age, admission during July–December, co-infection with Epstein-Barr virus, chlamydia, or other bacteria, and complications, including pleural effusion, abnormal liver enzymes, acute respiratory distress syndrome, or atelectasis, were associated with bronchoscopy. Older age, admission during October–December, co-infection with respiratory syncytial virus, adenovirus, Epstein-Barr virus, chlamydia, or other bacteria, and complications, including cardiac failure, abnormal liver enzymes, myocardial damage, or anemia, were associated with severe pneumonia. Cases of MPP, severe pneumonia, and those requiring bronchoscopy increased in 2023 compared with the five previous years. Children with MPP were more susceptible to adverse clinical outcomes, which were associated with age, month of admission, co-infection, and complications.

**IMPORTANCE:**

*Mycoplasma pneumoniae* pneumonia (MPP) is a leading cause of community-acquired pneumonia in children. This study analyzed 17,505 hospitalized children in Northeast China from 2018 to 2023 to explore MPP trends before, during, and after the coronavirus disease 2019 pandemic. We found a sharp resurgence of MPP in 2023 with higher rates of severe pneumonia, complications, and bronchoscopy use. Key risk factors for adverse outcomes included older age, admission in October–December, viral/bacterial co-infection, and pulmonary or extrapulmonary complications. These findings improve understanding of post-pandemic MPP epidemiology, support early risk stratification, and help optimize clinical management and public health interventions for childhood MPP.

## INTRODUCTION

*Mycoplasma pneumoniae* pneumonia (MPP) is the most common form of community-acquired pneumonia (CAP) (1, 2). Approximately 70% of children aged over 5 years become infected with *M. pneumoniae* (3). The epidemic interval of MPP worldwide is 3–7 years, accounting for more than 40% of pediatric CAP cases in epidemic years (2). A brief MPP epidemic occurred in Northeast China in 2019. However, following the subsequent outbreak of the coronavirus disease 2019 (COVID-19) at the end of 2019, the Chinese government adopted strong infection mitigation measures, which lasted until the end of 2022, blocking the epidemic of respiratory pathogens, including COVID-19, and also affecting the epidemic trend of MPP (4). At the end of 2022, the Chinese government lifted these strong mitigation measures. This policy shift was soon followed by an outbreak of COVID-19 over a short period of time, concomitant with changes in the prevalence of other respiratory pathogens. Notably, MPP in children emerged as a regional epidemic in 2023.

Although most children with MPP have mild clinical symptoms, a significant proportion experience adverse outcomes, especially during an epidemic (5, 6). Adverse outcomes during the MPP pandemic mainly manifested as an increase in the proportion of severe pneumonia and intrapulmonary and extrapulmonary complications. The frequency of use of corresponding special procedures and the number of pediatric intensive care unit (PICU) admissions also increased (7, 8). The concentrated occurrence of these adverse events can rapidly overwhelm healthcare system resources in a short period of time, increasing the healthcare burden.

In this study, we analyzed the data of children hospitalized with MPP in Northeast China before and during the COVID-19 pandemic from 2018 to 2023 to determine the prevalence of MPP, the complication rate, the probability of adverse outcomes, and the factors associated with adverse outcomes. These data will ultimately increase public attention to MPP and contribute to the protection of children’s health as well as the development of public health infrastructure, surveillance, and interventions.

## MATERIALS AND METHODS

### Study design

This retrospective cohort study included children with MPP who were treated in the pediatric ward of Shengjing Hospital of China Medical University, one of four National Children’s Regional Medical Centers, between January 2018 and December 2023. MP infection was confirmed through clinical symptoms and signs (fever, cough, or abnormal lung auscultation), increased chest X-ray infiltration, and positive laboratory results for MP. Nucleic acid amplification was performed on nasopharyngeal swabs or bronchoalveolar lavage fluid using real-time polymerase chain reaction (PCR) targeting MP-specific gene fragments (e.g., P1 gene, 16S rDNA, or 16S rRNA) (9). Alternatively, serological confirmation was based on a single serum antibody titer ≥1:160 (detected by particle agglutination assay) or seroconversion and/or a ≥4-fold antibody titer increase in acute and convalescent paired serum samples collected at an interval of ≥2 weeks (9).

Those with incomplete information (for example, on age, sex, or diagnosis) and those admitted to the neonatal intensive care unit were excluded, and the follow-up period lasted from admission to the final clinical outcome, including full recovery or death.

### Data collection

The medical records were reviewed to obtain information on demographic characteristics (including age, sex, and admission year and month), pathogenic examinations, underlying diseases (including chronic lung disease, congenital heart disease, neuromuscular disease, malnutrition, asthma, and anemia), intrapulmonary complications (including hypoxemia, respiratory failure, acute respiratory distress syndrome, pleural effusion, atelectasis, pneumothorax, and plastic bronchitis), extrapulmonary complications (including myocardial damage, cardiac failure, abnormal liver enzyme indicators, sepsis, skin involvement, arterial embolism, laryngitis, and encephalitis), medical procedures (including bronchoscopy, thoracentesis, closed thoracic drainage, plasma exchange, NO inhalation, extracorporeal membrane oxygenation [ECMO], lumbar puncture, oxygen supplementation, non-invasive mechanical ventilation, and invasive mechanical ventilation), and outcomes (including severe pneumonia, PICU admission, duration of hospital stay, death, and total and average costs).

Co-infection with other common respiratory pathogens, including respiratory syncytial virus (RSV), Epstein-Barr virus (EBV), adenovirus (Adv), influenza virus, chlamydia, COVID-19, and other bacteria, was detected using commercial multiplex PCR panels on nasopharyngeal swabs combined with routine bacterial culture of respiratory specimens.

Children were assigned to three groups based on the implementation of stringent COVID-19 prevention and control measures in China: pre-COVID-19 period (2018–2019); during COVID-19 period with controls (2020–2022), when stringent measures were implemented; and post-COVID-19 period without controls (2023), following the relaxation of these measures. Additional grouping variables included admission quarter, sex, co-infection, underlying disease, and complications. Children were divided into four groups according to age (0–1, 1–3, 3–6, and >6 years), with children aged 1 year included in the 1–3 group. All data were curated by a trained researcher.

### Outcome measures

The primary outcomes were the use of bronchoscopy and the development of severe pneumonia. The secondary outcomes were respiratory failure, hospital stay lasting more than 7.5 days, and total treatment costs per child of more than $1,255.81. In particular, severe pneumonia was defined as pneumonia accompanied by one of the following characteristics: (i) the general condition is poor, (ii) the respiratory rate increases, (iii) difficulty breathing and cyanosis, (iv) multilobed involvement or ≥2/3 lung involvement, (v) extrapulmonary complications, (vi) pleural effusion, and (vii) percutaneous oxygen saturation in indoor air ≤92% (10).

### Statistical analysis

Categorical variables are expressed as numbers and proportions and were compared between groups using Pearson’s chi-square test. The total cost was calculated as the sum of all hospitalization expenses for each year, while the average cost was calculated by dividing the total cost by the number of children treated per year. To identify factors associated with the outcome measures, multivariable logistic regression analysis was conducted using age, sex, admission quarter, and complications as covariates. Statistical significance was set at *P* < 0.05. SPSS software version 27.0 (IBM Corporation, Armonk, NY, USA) was used for data analysis, while R software version 4.2.0 (R Foundation for Statistical Computing, Vienna, Austria), GraphPad Prism 8.0 (GraphPad Inc., La Jolla, CA, USA), and Origin 2024 (OriginLab Corporation, Northampton, MA, USA) were used to create figures.

## RESULTS

### Demographic and clinical characteristics of children with MPP

**Fig 1:**
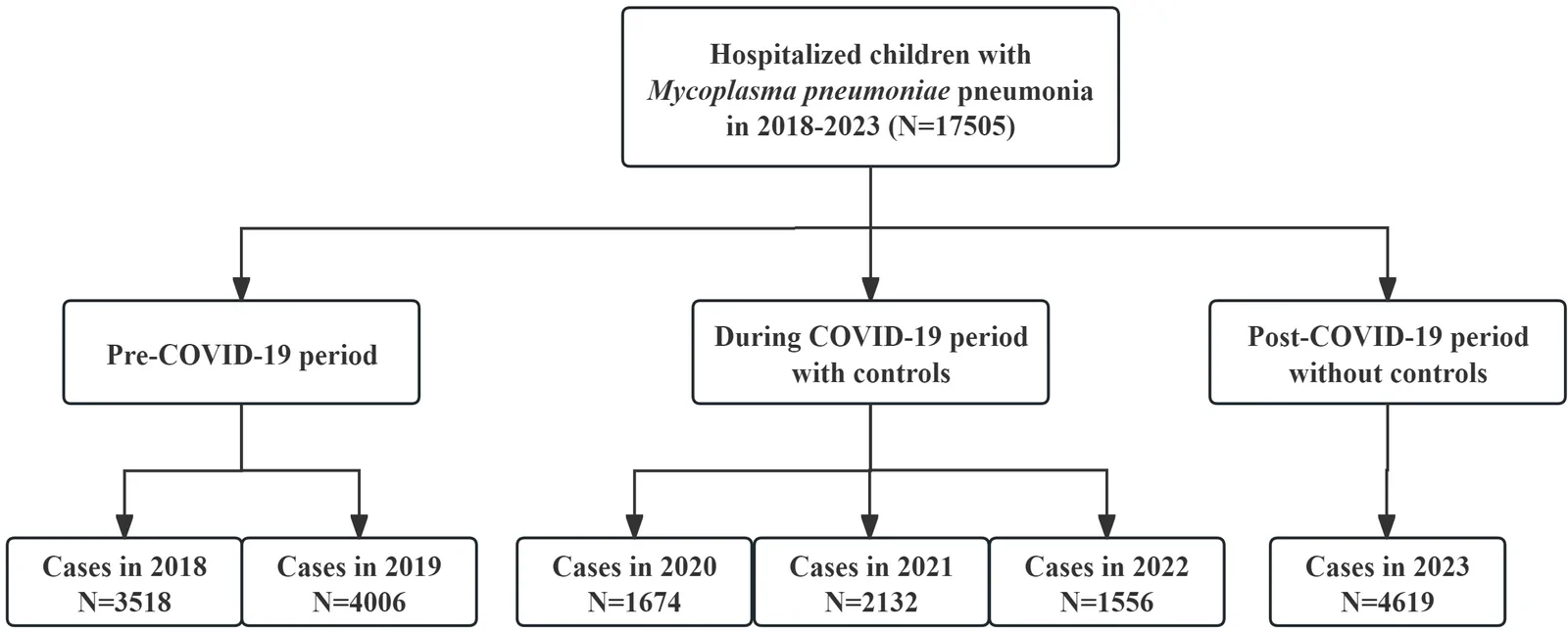
Flow chart of the study design.

From 2018 to 2023, 17,505 children aged 28 days to 14 years were hospitalized due to MPP, all of whom had complete clinical data and were therefore included in the MPP cohort. The total number of MPP cases by year was 3,518 in 2018, 4,006 in 2019, 1,674 in 2020, 2,132 in 2021, 1,556 in 2022, and 4,619 in 2023 (Fig. 1). EBV was the most commonly detected co-infection among children hospitalized due to MPP (*P* < 0.001) (Table 1).

**TABLE 1:** Demographic and clinical characteristics of the enrolled children with *Mycoplasma pneumoniae* pneumonia^*a*^^,*b*^

Characteristic	Pre-COVID-19 period		During COVID-19 period with controls			Post-COVID-19 period without controls	
	2018	2019	2020	2021	2022	2023	*P* value
	*n* = 3,518	*n* = 4,006	*n* = 1,674	*n* = 2,132	*n* = 1,556	*n* = 4,619	
Age (years)							<0.01
0–1	322 (9.2)	397 (9.9)	281 (16.8)	255 (12.0)	202 (13.0)	220 (4.8)	
1–3	1,464 (41.6)	2,070 (51.7)	877 (52.4)	1,328 (62.3)	607 (39.0)	932 (20.2)	
3–6	1,017 (28.9)	923 (23.0)	265 (15.8)	374 (17.5)	402 (25.8)	1,247 (27.0)	
>6	715 (20.3)	616 (15.4)	251 (15.0)	175 (8.2)	345 (22.2)	2,220 (48.1)	
Sex							0.198
Male	1,810 (51.4)	1,991 (49.7)	881 (52.6)	1,065 (50.0)	811 (52.1)	2,388 (51.7)	
Female	1,708 (48.6)	2,015 (50.3)	793 (47.4)	1,067 (50.0)	745 (47.9)	2,231 (48.3)	
Ethnicity							<0.01
Han	2,826 (80.3)	3,250 (81.1)	1,320 (78.9)	1,618 (75.9)	1,208 (77.6)	3,558 (77.0)	
Non-Han	692 (19.7)	756 (18.9)	354 (21.1)	514 (24.11)	348 (22.4)	1,061 (23.0)	
Quarter							<0.01
January–March	785 (22.3)	833 (20.8)	638 (38.1)	343 (16.1)	505 (32.5)	466 (10.1)	
April–June	631 (17.9)	776 (19.3)	96 (5.7)	775 (36.4)	128 (8.2)	549 (11.9)	
July–September	1,124 (31.9)	1,196 (29.9)	368 (22)	528 (24.8)	496 (31.9)	1,286 (27.8)	
October–December	978 (27.8)	1,201 (30.0)	572 (34.2)	580 (27.2)	395 (25.4)	2,318 (50.2)	
Co-infected with other pathogens							
RSV	52 (1.5)	184 (4.6)	63 (3.8)	121 (5.7)	45 (2.9)	139 (3.0)	<0.01
EBV	478 (13.6)	693 (17.3)	252 (15.1)	369 (17.3)	332 (21.3)	823 (17.8)	<0.01
Adv	60 (1.7)	217 (5.4)	67 (4.0)	27 (1.3)	8 (0.5)	199 (4.3)	<0.01
Influenza virus	60 (1.7)	120 (3.0)	6 (0.4)	13 (0.6)	29 (1.9)	93 (2.0)	<0.01
Chlamydia	147 (4.2)	245 (6.1)	86 (5.1)	112 (5.3)	65 (4.1)	219 (4.7)	0.002
COVID-19 virus	–	–	–	–	1 (0.06)	17 (0.4)	0.055
Other bacteria	43 (1.2)	4 (0.1)	37 (2.2)	68 (3.2)	60 (3.9)	147 (3.2)	<0.01
Underlying disease							
Chronic pulmonary disease	7 (0.2)	12 (0.3)	6 (0.4)	2 (0.09)	2 (0.1)	10 (0.2)	<0.01
Congenital heart disease	25 (0.7)	22 (0.5)	23 (1.4)	7 (0.3)	4 (0.3)	51 (1.1)	<0.01
Neuromuscular disease	116 (3.3)	110 (2.7)	27 (1.6)	14 (0.7)	14 (0.9)	178 (3.9)	<0.01
Malnutrition	8 (0.2)	7 (0.2)	2 (0.1)	1 (0.05)	5 (0.3)	3 (0.6)	<0.01
Asthma	354 (10.1)	500 (12.5)	307 (18.3)	359 (16.8)	239 (15.4)	231 (5.0)	<0.01
Anemia	93 (2.6)	128 (3.2)	91 (5.4)	44 (2.1)	25 (1.6)	141 (3.1)	<0.01
Intrapulmonary complications							
Hypoxemia	172 (4.9)	245 (6.1)	144 (8.6)	102 (4.8)	103 (6.6)	334 (7.2)	<0.01
Respiratory failure	32 (0.9)	65 (1.6)	44 (2.6)	40 (1.9）	28 (1.8)	125 (2.7)	<0.01
Respiratory distress syndrome	9 (0.3)	7 (0.2)	–	2 (0.1)	3 (0.2)	33 (0.7)	<0.01
Pleural effusion	250 (7.1)	255 (6.4)	57 (3.4)	48 (2.3)	77 (4.9)	675 (14.6)	<0.01
Atelectasis	6 (0.2)	6 (0.1)	9 (0.5)	7 (0.3)	22 (1.4)	71 (1.5)	<0.01
Pneumothorax	8 (0.2)	4 (0.1)	8 (0.5)	5 (0.2)	6 (0.4)	15 (0.3)	0.117
Plastic bronchitis	12 (0.3)	15 (0.4)	5 (0.3)	2 (0.09)	14 (0.9)	61 (1.3)	<0.01
Extrapulmonary complications							
Myocardial damage	45 (1.3)	43 (1.1)	–	2 (0.1)	4 (0.3)	51 (1.1)	<0.01
Cardiac failure	6 (0.2)	4 (0.1)	4 (0.2)	6 (0.3)	3 (0.2)	3 (0.06)	0.180
Abnormal liver enzyme indicators	136 (3.9)	162 (4.0)	95 (5.7)	51 (2.4)	8 (0.5)	133 (2.9)	<0.01
Sepsis	108 (3.1)	298 (7.4)	173 (10.3)	224 (10.5)	78 (5.0)	134 (2.9)	<0.01
Skin involvement	38 (1.1)	28 (0.7)	27 (1.6)	27 (1.3)	14 (0.9)	58 (1.3)	<0.01
Arterial embolism	–	3 (0.07)	–	1 (0.05)	3 (0.2)	23 (0.5)	<0.01
Laryngitis	–	2 (0.05)	3 (0.2)	–	–	1 (0.02)	0.055
Encephalitis	9 (0.3)	19 (0.5)	10 (0.6)	13 (0.6)	14 (0.9)	180 (3.9)	<0.01

^*a*^ Data are presented as numbers (%) and were compared using Pearson’s chi-square test.

^*b*^ Adv, adenovirus; EBV, Epstein-Barr virus; and RSV, respiratory syncytial virus; –, no data available.

### Patient age

From 2018 to 2022, the proportion of hospitalized children aged 1–3 years was significantly higher than that of other age groups (41.6% in 2018, 51.7% in 2019, 52.4% in 2020, 62.3% in 2021, and 39% in 2022). However, in 2023, the highest proportion of patients (48.1%) were in the >6 years age group (Table 1; Fig. 3A).

### Month of admission

**Fig 2:**
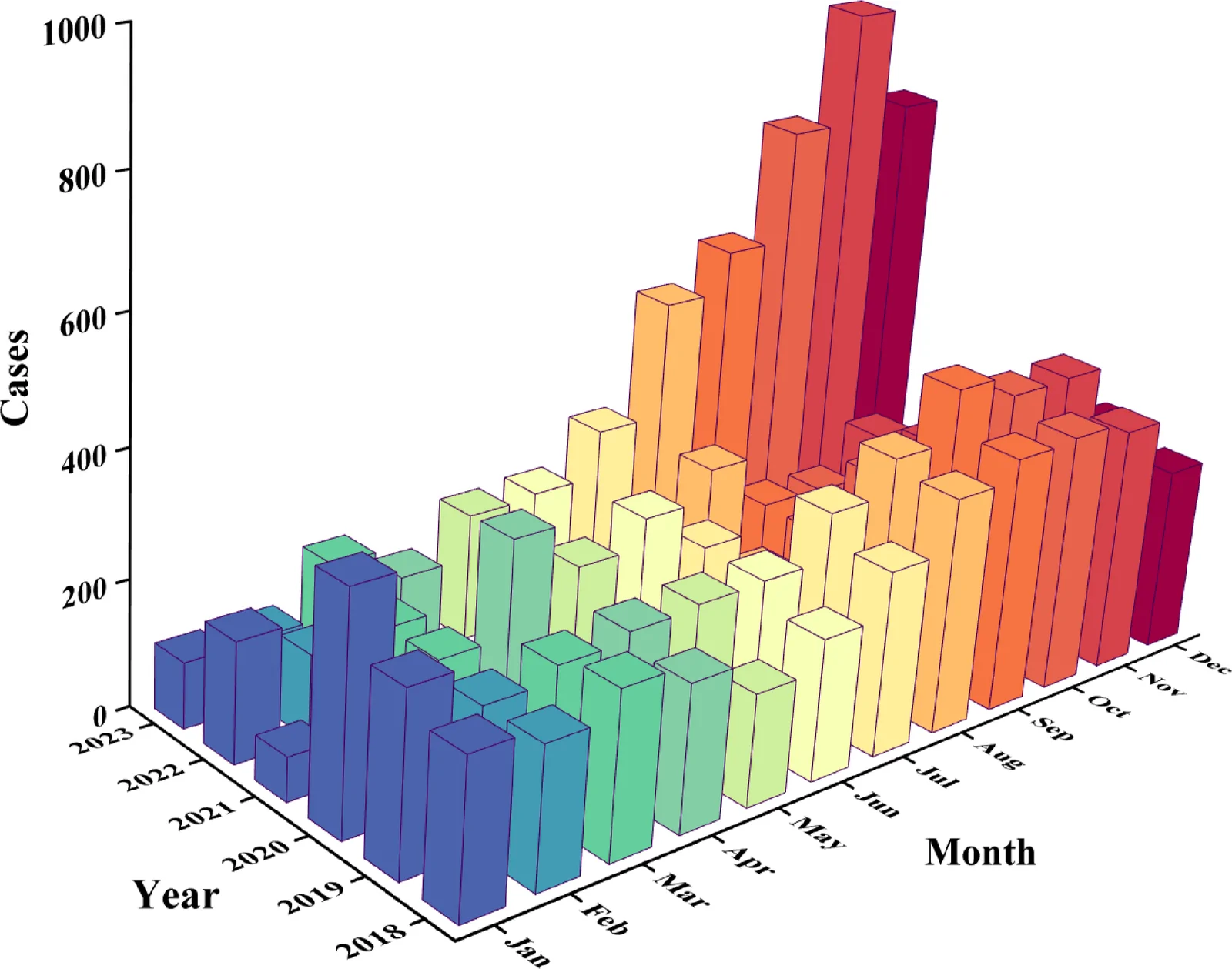
Trends in MPP cases from 2018 to 2023 according to month of admission.

**Fig 3:**
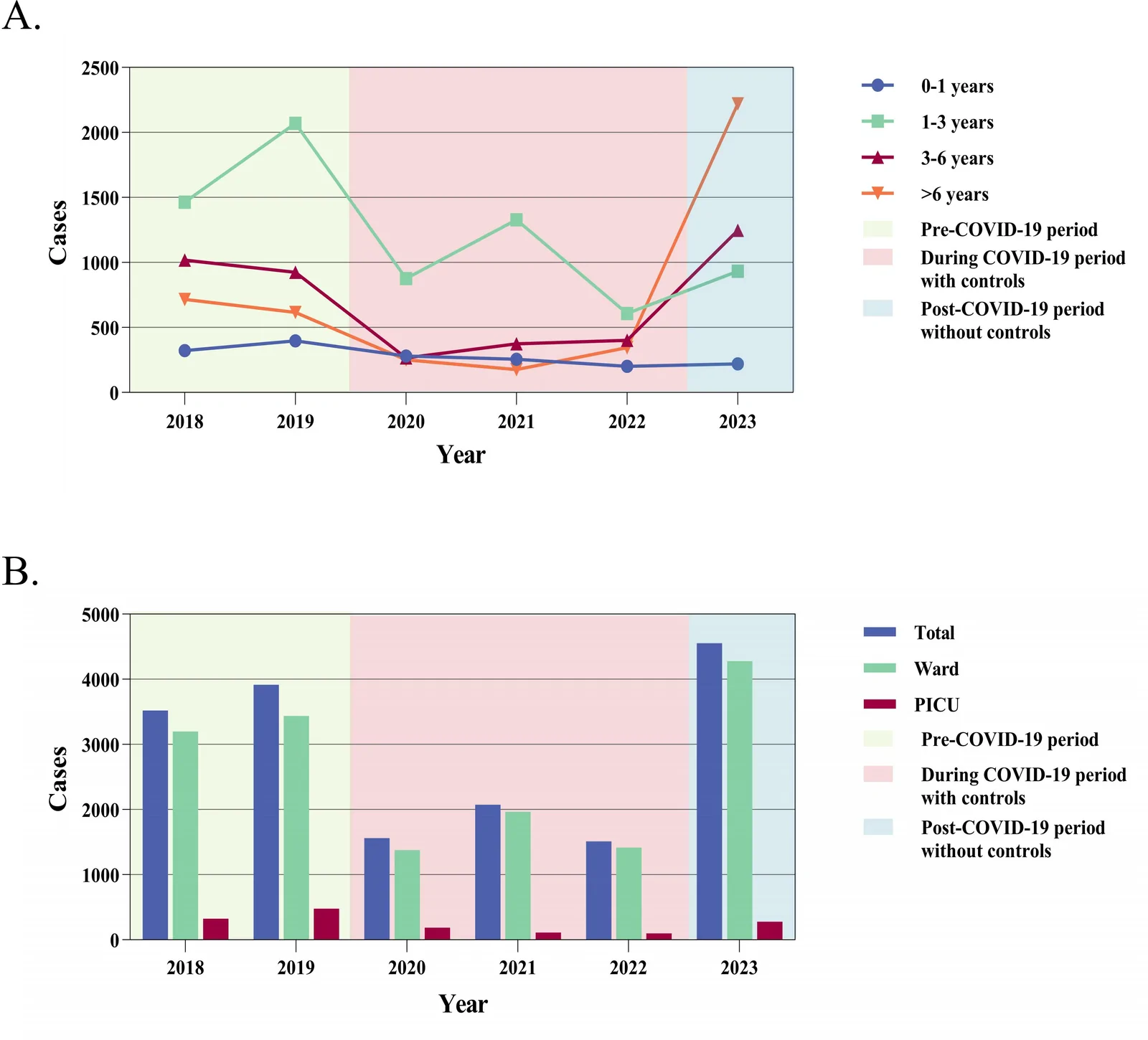
Trends in MPP cases from 2018 to 2023 according to (**A**) patient age and (**B**) ward type. Coronavirus disease 2019, COVID-19; PICU, pediatric intensive care unit.

In 2023, hospital admissions were highest from October to December (*n* = 2,318), increasing gradually throughout the year and peaking in November. From 2018 to 2023, hospital admissions increased between September and November, although to a much lesser extent than in 2023, showing a downward trend after November (Table 1; Fig. 2). In terms of ward type, the number of patients admitted to the PICU was the highest in 2019 (Table 2; Fig. 3B).

**TABLE 2:** Procedural interventions, respiratory support, and outcomes of children with *Mycoplasma pneumoniae* pneumonia^*c*^^,^^*d*^

	2018	2019	2020	2021	2022	2023	*P* value
	*n* = 3,518	*n* = 4,006	*n* = 1,674	*n* = 2,132	*n* = 1,556	*n* = 4,619	
Procedural interventions							
Bronchoscopy	346 (9.8)	304 (7.6)	121 (7.2)	120 (5.6)	185 (11.9)	1,547 (33.5)	<0.01
Thoracocentesis	18 (0.5)	27 (0.7)	20 (1.2)	10 (0.5)	3 (0.2)	49 (1.1)	<0.01
Closed thoracic drainage	6 (0.2)	6 (0.1)	4 (0.2)	5 (0.2)	2 (0.1)	9 (0.2)	<0.01
Plasma exchange	2 (0.06)	9 (0.2)	6 (0.4)	12 (0.6)	5 (0.3)	10 (0.2)	<0.01
NO inhalation	2 (0.06)	–	–	–	–	7 (0.2)	<0.01
ECMO	1 (0.03)	3 (0.07)	1 (0.06)	1 (0.05)	1 (0.06)	4 (0.09)	<0.01
Lumbar puncture	36 (1.0)	61 (1.5)	43 (2.7)	42 (2.0)	29 (1.9)	107 (2.3)	<0.01
Respiratory support							
Oxygen supplementation	178 (5.1)	262 (6.5)	144 (8.6)	103 (4.8)	101 (6.5)	366 (7.9)	<0.01
Non-invasive mechanical ventilation	10 (0.3)	17 (0.4)	21 (1.3)	11 (0.5)	10 (0.6)	30 (0.6)	<0.01
Invasive mechanical ventilation	16 (0.5)	31 (0.8)	23 (1.4)	28 (1.3)	20 (1.3)	63 (1.4)	<0.01
Outcomes							
Severe pneumonia	561 (15.9)	711 (17.7)	233 (13.9)	175 (8.2)	282 (18.1)	2,038 (44.1)	<0.01
PICU admission	322 (9.2)	478 (11.9)	185 (11.1)	109 (5.1)	96 (6.2)	276 (6.0)	<0.01
Duration of hospitalization	8 (7–9)	8 (6–9)	8 (6–10)	7 (6–9)	7 (6–9)	7 (6–9)	<0.01
Deaths	8 (0.2)	2 (0.04)	4 (0.2)	4 (0.2)	1 (0.06)	1 (0.02)	<0.01
Total cost (×10^6^ $)^*a*^	5.89	7.34	3.13	2.96	2.51	6.78	NA
Average cost (×10^3^ $)^*b*^	1.67	1.83	1.87	1.39	1.61	1.47	NA

^*a*^ Sum of all hospitalization expenses for each year.

^*b*^ Calculated by dividing the total cost by the number of children treated per year.

^*c*^ Data are presented as numbers (%) and compared using Pearson’s chi-square test unless otherwise specified.

^*d*^ PICU, pediatric intensive care unit; NO, nitric oxide; ECMO, extracorporeal membrane oxygenation; NA, not applicable; –, no data available.

### Complications

In 2023, the proportion of children with MPP complicated by intrapulmonary and extrapulmonary complications increased significantly (*P* < 0.001) (Table 1).

### Procedural interventions, respiratory support, and outcomes of children with MPP

The probability of developing severe pneumonia was 44.1% in 2023, which was significantly higher than that in 2018–2022. In addition to pharmacotherapy, including macrolides, tetracyclines, and glucocorticoids, various procedural interventions and respiratory support measures were applied in a subset of patients. Among procedural interventions, the utilization rates of bronchoscopy (33.5%) and thoracocentesis (1.1%) were significantly higher in 2023 than in 2018–2022. Regarding respiratory support, the proportions of patients receiving oxygen therapy (8.6%) and non-invasive mechanical ventilation (1.3%) were highest in 2020 among the six study years. Eight and two patients admitted in 2018 and 2019, respectively, died, four each in 2020 and 2021, and one each in 2022 and 2023 (Table 2).

**Fig 4:**
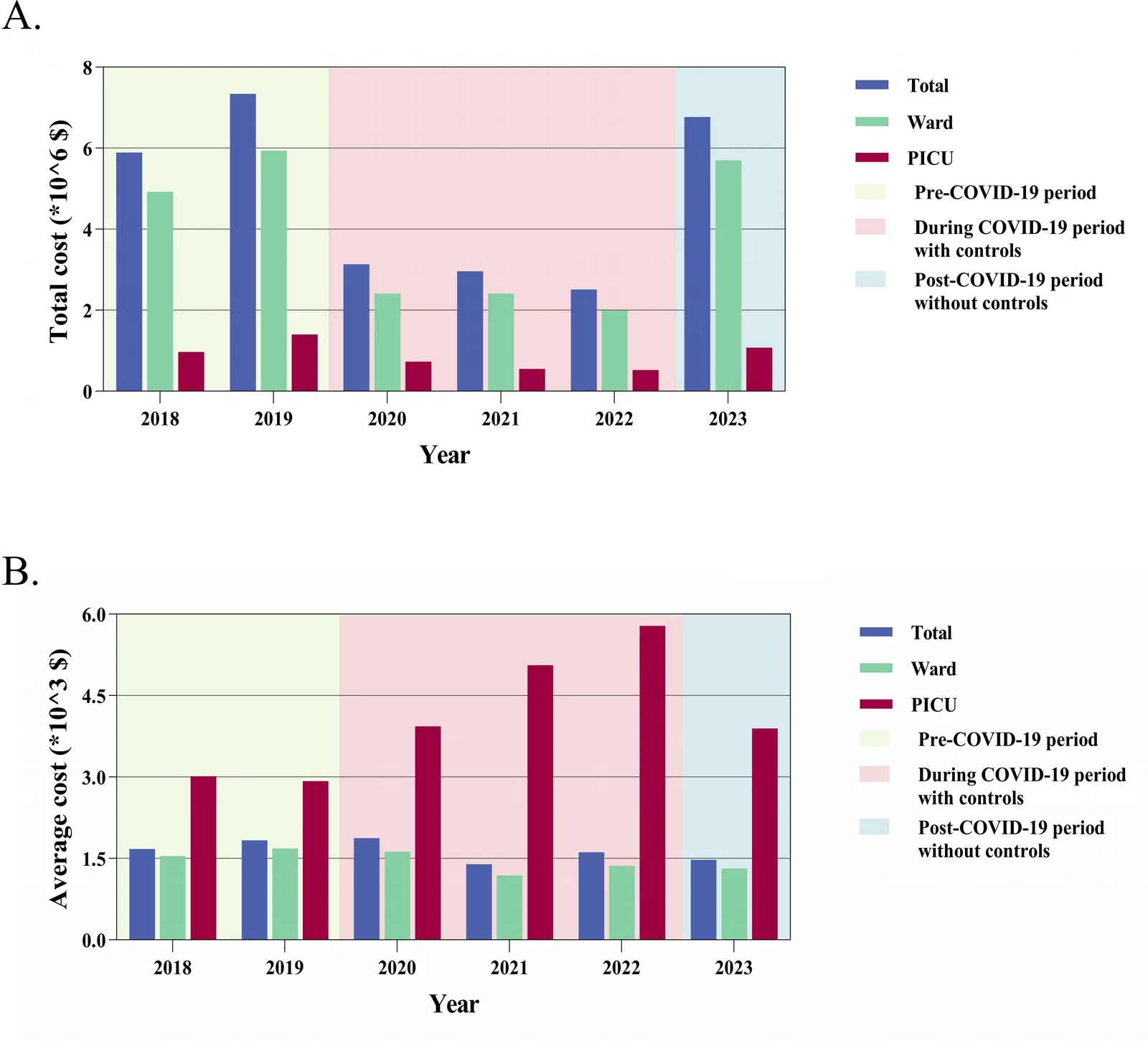
Trends in the distribution of (A) total and (B) average hospitalization costs. PICU, pediatric intensive care unit.

In general wards, medical service fees were responsible for the highest proportion of costs, at 30% (Table S2). Conversely, in the PICU, other expenses accounted for 28%, whereas medical service fees and medication accounted for 25% (Table S2). From 2020 to 2022, other expenses were responsible for the highest proportion of the total expenditure, reaching more than 30%. In 2023, medical service fees accounted for the highest proportion of total expenditure at 31% (Table S3). Total hospitalization expenses were highest in 2019 ($7.34 × 10^6^), with PICU-associated costs of $1.40 × 10^6^. Average PICU-associated costs were highest in 2022, at $5.78 × 10^3^ (Fig. 4A and B).

**Fig 5:**
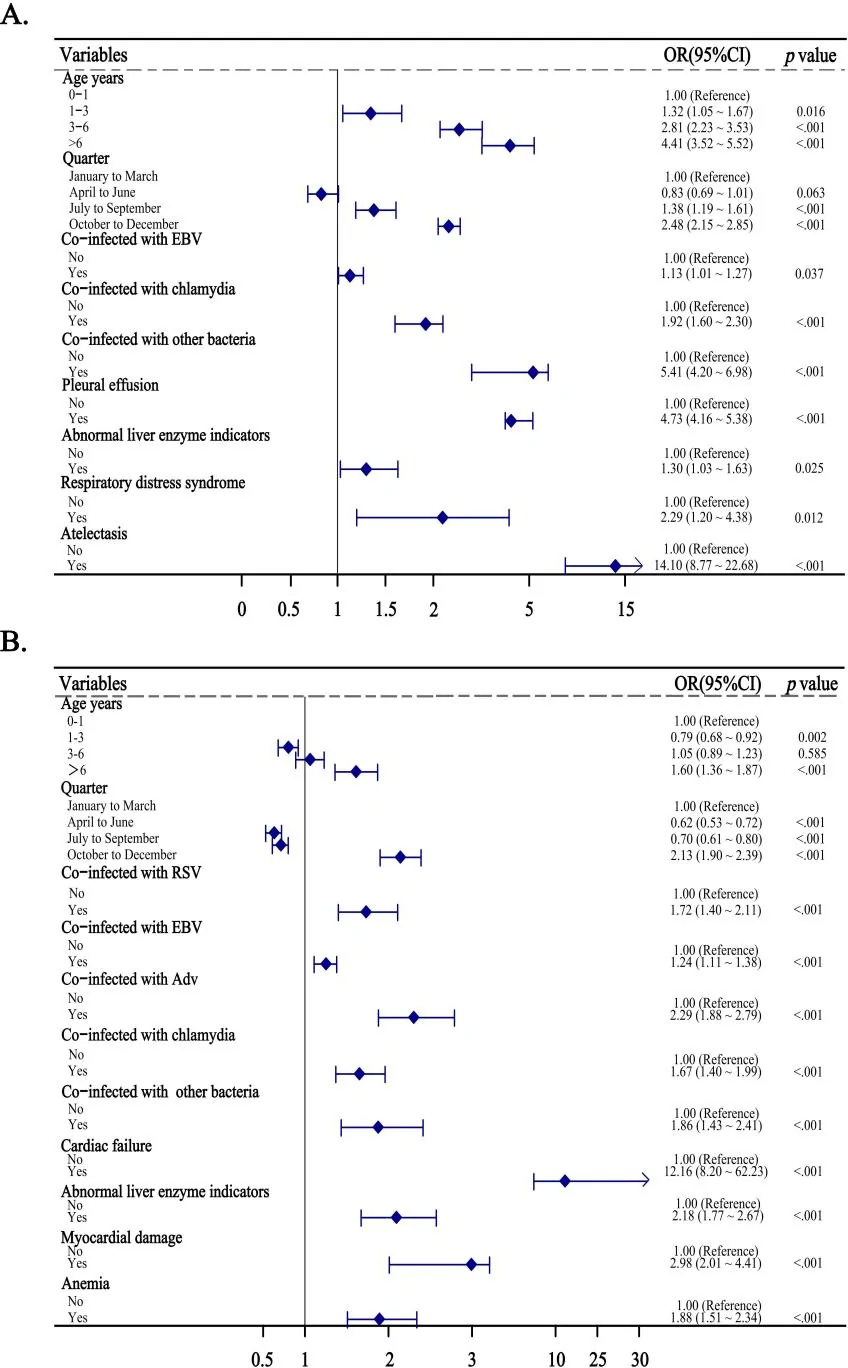
Factors associated with (**A**) bronchoscopy and (**B**) severe pneumonia. Adv, adenovirus; CI, confidence interval; EBV, Epstein-Barr virus; RSV, respiratory syncytial virus; and OR, odds ratio.

In the present study, logistic regression analysis was performed to identify factors associated with the primary outcomes of bronchoscopy and severe pneumonia (Fig. 5), as well as the secondary outcomes of respiratory failure, hospitalization time >7.5 days, and hospitalization expenses >$1,255.81 (Table S1). The risk of undergoing bronchoscopy was significantly increased in patients aged 1–3 years (odds ratio [OR], 1.32; 95% confidence interval [CI], 1.05,1.67), 3–6 years (OR, 2.81; 95% CI, 2.23,3.53), or >6 years (OR, 4.41; 95% CI, 3.52,5.52) compared with those aged 0–1 years, as well as in those admitted to hospital between July and September (OR, 1.38; 95% CI, 1.19,1.61) or October and December (OR, 2.48; 95% CI, 2.15,2.85) than in those admitted between January and March. Co-infection with EBV, chlamydia, or other bacteria also significantly increased the risk of requiring bronchoscopy for MPP, as did developing the complications of pleural effusion, abnormal liver enzyme indicators, respiratory distress syndrome, or atelectasis (Fig. 5A).

The risk of developing severe pneumonia was significantly decreased in patients aged 1–3 years (OR, 0.79; 95% CI, 0.68,0.92) and significantly increased in patients aged >6 years (OR, 1.60; 95% CI, 1.36,1.87) compared with those aged 0–1 years. The risk of severe pneumonia was significantly decreased in patients admitted between April and June (OR, 0.62; 95% CI, 0.53,0.72) or July and September (OR, 0.70; 95% CI, 0.61,0.80) but significantly increased in patients admitted between October and December (OR, 2.13; 95% CI, 1.90,2.39) compared with those admitted between January and March. Co-infection with RSV, EBV, Adv, chlamydia, or other bacteria also significantly increased the risk of severe pneumonia, as did cardiac failure, abnormal liver enzyme indicators, myocardial damage, or anemia (Fig. 5B).

### Characteristics and prognosis in children with MPP co-infected with COVID-19

Although COVID-19 first emerged in 2019, strict control measures prevented its widespread transmission in northeast China until the end of 2022. During the first outbreak of COVID-19, there were a total of 18 hospitalized children with MPP co-infected with COVID-19 (1 case in 2022 and 17 cases in 2023), including 7 critically ill patients hospitalized in the PICU (38.9%), yielding a PICU admission rate higher than that of MPP patients without COVID-19 (8.4%).

## DISCUSSION

In recent years, the incidence and severity of MPP have fluctuated worldwide, showing an overall upward trend (11). MPP is a cyclical disease, with cycles lasting 3–7 years and prevalence peaking approximately every 4.5 years, a cycle that may be associated with population-level changes in immunity (12–14). A new round of outbreaks caused by drug-resistant *M. pneumoniae* clones was predicted to occur around 2020. Consistent with this, our study showed an increased incidence of MPP in the third and fourth quarters of 2019. However, the outbreak of COVID-19, first reported in China in December 2019, resulted in the implementation of various infection prevention and control measures, including stay-at-home orders, strict social distancing, and the wearing of masks, over the following 3 years (15). These instructions, combined with a general improvement in public awareness of respiratory infection prevention and the concentrated investment of medical resources toward COVID-19, led to a decreased incidence of MPP, with the number of patients treated annually only approximately half of those in the pre-COVID-19 years (16). These results indicate that the emergence of COVID-19 delayed the MPP cycle, and a large-scale outbreak could therefore have been expected to occur in 2023.

The present study revealed that the number of MPP cases in 2023 was higher than in several preceding years, including those in the years prior to the COVID-19 pandemic. Moreover, children with MPP developed more severe outcomes, including more severe intrapulmonary and extrapulmonary complications, a higher incidence of severe pneumonia, a greater requirement for procedures such as bronchoscopy, a higher PICU admission rate, and higher total costs. We speculate that this may be explained by the following: long-term low exposure to *Mycoplasma* pathogens led to insufficient protective immunity and a larger proportion of the population being susceptible to MPP, a situation known as immune debt or immunity gap; social isolation and COVID-19 damaged immune systems, causing a decline in resistance to external pathogens; and the emergence of macrolide-resistant *M. pneumoniae* (MRMP) (17, 18). These conditions, combined with the cyclical nature of MPP, led to an explosive outbreak of MPP in 2023, which is ongoing.

MPP in children accounts for 10%–40% of all CAP, while children aged 1–3 years are most susceptible to CAP (19). Previous studies have shown that school-age children and adolescents are more susceptible to MPP (11). However, in our study, children aged 1–3 years still accounted for the largest proportion of cases from 2018 to 2022, whereas children aged >6 years accounted for the largest proportion of cases only in 2023. This may be because only children admitted to the hospital were included in this study, and children aged 1–3 years are more likely to be hospitalized and develop severe pneumonia than older children.

In addition, our study revealed that although MPP occurred year-round, its incidence was highest in the third and fourth quarters, which is consistent with the findings of previous studies (11). As our study was conducted in the cold climate of Northeast China, the seasonal prevalence pattern of MPP may differ from that of central or tropical regions. Indeed, one study in the United States showed that the incidence of MPP peaks in late summer or early autumn, indicating regional variations in MPP prevalence (20).

The most common co-infection detected in the present study was EBV. In clinical practice, EBV infection commonly results in more serious symptoms and outcomes, which may be related to the EBV-induced activation of specific immune mechanisms (21–23). Following initial infection, EBV enters a latent state within B cells to avoid immunosurveillance, whereupon it subsequently undergoes a subclinical reactivation stage (21–23). As a result, the infection lasts longer, and the condition becomes more severe (21–23). In addition, although children infected with COVID-19 have milder clinical symptoms compared to adults, we found that children co-infected with COVID-19 had a higher rate of PICU admission than those without (24). However, as only 18 children with MPP were co-infected with COVID-19, there was no significant impact on the overall population’s medical resource utilization in this study.

*M. pneumoniae* infections exhibit a certain degree of self-limitation, with most cases being mild and associated with a favorable prognosis (25). However, some patients may progress to pneumonia or develop severe disease accompanied by pulmonary and/or extrapulmonary complications, and in severe cases, death may occur (25). In our study, 20 children hospitalized with MPP died between 2018 and 2023. Macrolide antibiotics are the preferred medication for MPP in children (25). Owing to their extensive use, MRMP has emerged and spread globally for over 20 years, with prevalence rates exceeding 90% in China (4, 26, 27). Studies suggest that tetracyclines and fluoroquinolones may be used as second-line therapies for the treatment of pediatric MRMP (28). In our center, macrolides remained the first-line therapy for initial MPP cases. For patients who failed to show clinical improvement after 72 h of macrolide treatment, we considered the possibility of MRMP and shifted to second-line antibiotics, such as tetracyclines or fluoroquinolones. Systemic glucocorticoids were added for severe or critical cases. Bronchoscopy was performed early in severely ill children suspected of having mucus plug obstruction or plastic bronchitis. Intravenous immunoglobulin was reserved for cases with severe extrapulmonary complications such as encephalitis, severe mucocutaneous lesions, or hematological manifestations.

Although the number of MPP cases was lower in 2019 than in 2023, the total and average costs were higher in 2019 than in 2023, which we speculate may have been related to the changes in medical insurance policies in China. In 2017, the National Health Insurance Bureau of China proposed to start the reform of diagnosis-related group (DRG) payment, while in 2020, it conducted simulation experiments in 30 cities in China (29). In 2021, the payment reform, DRG payment policy, was implemented in Northeast China, aiming to reduce the average hospitalization cost of inpatients (24, 29). The transformation of hospital operation mode in China was vigorously promoted from focusing on expanding income to focusing on cost control and optimization (30). The aforementioned results largely indicated that reasonable medical insurance policies have had a significant positive impact on the healthcare system. In addition, in 2023, the incidence of intrapulmonary complications, such as pleural effusion, and extrapulmonary complications, such as encephalitis, increased compared to previous years, leading to a higher demand for procedures such as pleural puncture, lumbar puncture, plasma exchange, and mechanical ventilation. At the same time, the proportion of patients with atelectasis, pulmonary consolidation, and plastic bronchitis was higher in 2023 than in any of the previous 5 years; as these are indications for bronchoscopy, a higher proportion of patients underwent bronchoscopies in 2023. Thus, the proportion of medical expenses due to medical service fees peaked in 2023.

Our study has certain limitations. First, it was a retrospective cohort study. Second, it was a single-center study. However, as the study was conducted at one of four National Children’s Regional Medical Centers in China, the patients should accurately represent the characteristics of children with MPP in Northeast China. Finally, we focused only on hospitalized children and did not analyze children with MPP in the community.

In summary, the present study found that more children were hospitalized due to MPP in 2023 than in any of the previous 5 years, and these children had higher rates of intrapulmonary and extrapulmonary complications, higher rates of severe pneumonia and PICU admission, greater requirement for specialist procedures, and relatively high total and average costs. Importantly, our study is the first to identify factors associated with bronchoscopy and severe pneumonia in children hospitalized due to MPP, including age, month of admission, co-infection with EBV, chlamydia, or other bacteria, and presence of complications of pleural effusion, liver dysfunction, respiratory distress syndrome, or atelectasis. These factors could potentially be used to stratify the risk of adverse outcomes in patients with MPP, allowing close monitoring or early intervention.

## Supplementary Material

Reviewer comments

## Data Availability

All data supporting the study findings are available from the corresponding author upon request.

## SUPPLEMENTAL MATERIAL

Tables S1 to S3 spectrum.00354-26-s0001.docx Table S1: Univariate logistic regression analysis of factors associated with respiratory failure, duration of hospital stay, and total treatment cost. Table S2: Trends in the distribution of costs according to ward type. Table S3: Trends in the distribution of costs according to year.

OPEN PEER REVIEW reviewer-comments.pdf An accounting of the reviewer comments and feedback.

ASM does not own the copyrights to Supplemental Material that may be linked to, or accessed through, an article. The authors have granted ASM a non-exclusive, world-wide license to publish the Supplemental Material files. Please contact the corresponding author directly for reuse.
