# Supplementary material for: Trends and outcomes of Mycoplasma pneumoniae pneumonia in hospitalized children before, during, and after the coronavirus disease 2019 pandemic: a retrospective study in Northeast China, 2018–2023
Source: Microbiol Spectr. 2026 Aug 14;14(9):e00354-26. doi: 10.1128/spectrum.00354-26 (PMC13532259; doi:10.1128/spectrum.00354-26)
Supplement: Supplementary file 1 [file reviewer-comments.pdf]

ASM | Microbiology Spectrum

Trends and outcomes of *Mycoplasma pneumoniae* pneumonia in hospitalized children before, during and after the coronavirus disease 2019 pandemic: A retrospective study in Northeast China, 2018–2023

Chunxue Jiang, Xin Chen, Xuewen Xu, TingTing Sun, Wei Xu, and Kai You

Corresponding Author(s): Kai You, Shengjing Hospital of China Medical University

**Review Timeline:**		
	Submission Date:	January 27, 2026
	Editorial Decision:	March 9, 2026
	Revision Received:	May 8, 2026
	Editorial Decision:	June 22, 2026
	Revision Received:	June 30, 2026
	Accepted:	July 24, 2026

Editor: Cecilia Thompson

Reviewer(s): Disclosure of reviewer identity is with reference to reviewer comments included in decision letter(s). The following individuals involved in review of your submission have agreed to reveal their identity: Leslie A Wolf (Reviewer #1); Sreenath Karekatt (Reviewer #2)

Transaction Report:

(Note: With the exception of the correction of typographical or spelling errors that could be a source of ambiguity, letters and reports are not edited. The original formatting of letters and referee reports may not be reflected in this compilation.)

DOI: https://doi.org/10.1128/spectrum.00354-26

Re: Spectrum00354-26 (**Trends and outcomes of *Mycoplasma pneumoniae* pneumonia in hospitalized children before and during the coronavirus disease 2019 pandemic: A retrospective study in Northeast China, 2018-2023**)

Dear Ms. Chunxue Jiang:

Thank you for the privilege of reviewing your work. Below you will find my comments, instructions from the Spectrum editorial office, and the reviewer comments.

Please return the manuscript within 60 days; if you cannot complete the modification within this time period, please contact me. If you do not wish to modify the manuscript and prefer to submit it to another journal, notify me immediately so that the manuscript may be formally withdrawn from consideration by Spectrum.

Revision Guidelines
To submit your modified manuscript, log into the submission site at https://spectrum.msubmit.net/cgi-bin/main.plex. Go to Author Tasks and click the appropriate manuscript title to begin. The information you entered when you first submitted the paper will be displayed; update this as necessary. Note the following requirements:

• Upload point-by-point responses to the issues raised by the reviewers in a file named "Response to Reviewers," NOT in your cover letter.
• Upload a compare copy of the manuscript (without figures) as a "Marked-Up Manuscript" file.
• Upload a clean .DOC/.DOCX version of the revised manuscript and remove the previous version.
• Each figure must be uploaded as a separate, editable, high-resolution file (TIFF or EPS preferred), and any multipanel figures must be assembled into one file.
• Any supplemental material intended for posting by ASM should be uploaded with their legends separate from the main manuscript. You can combine all supplemental material into one file (preferred) or split it into a maximum of 10 files with all associated legends included.

For complete guidelines on revision requirements, see our Submission and Review Process webpage. Submission of a paper that does not conform to guidelines may delay acceptance of your manuscript.

Data availability: ASM policy requires that data be available to the public upon online posting of the article, so please verify all links to sequence records, if present, and make sure that each number retrieves the full record of the data. If a new accession number is not linked or a link is broken, provide Spectrum production staff with the correct URL for the record. If the accession numbers for new data are not publicly accessible before the expected online posting of the article, publication may be delayed; please contact production staff (Spectrum@asmusa.org) immediately with the expected release date.

Publication Fees: For information on publication fees and which article types are subject to charges, visit our website. If your manuscript is accepted for publication and any fees apply, you will be contacted separately about payment during the production process; please follow the instructions in that e-mail. Arrangements for payment must be made before your article is published.

ASM Membership: Corresponding authors may join or renew ASM membership to obtain discounts on publication fees. Need to upgrade your membership level? Please contact Customer Service at Service@asmusa.org.

The ASM Journals program strives for constant improvement in our submission and publication process. Please tell us how we can improve your experience by taking this quick Author Survey.

Thank you for submitting your paper to Spectrum.

Sincerely,
Cecilia Thompson
Editor
Microbiology Spectrum

Reviewer #1 (Comments for the Author):

Review of Manuscript # Spetrum00354-26, "Trends and Outcomes of Mycoplasma pneumoniae pneumonia in hospitalized children before and during the coronavirus diseases 2019 pandemic: A retrospective study in Northeast China, 2018-2023"
• General comments:

o The manuscript is concise and organized logically so that it is easy to follow. One general comment is that Mycoplasma pneumoniae is not italicized throughout the document and figures/tables so this should be corrected.
o Specific comments include the following:
 Line 39 of the Abstract has a random "44" at the end of conclusions.
 Lines 49-53 seems to be a run on sentence that could be reworked for ease of reading.
 Line 53 and 55 refer to MPP as a pandemic. Please cite a reference for this or revise to say regional outbreak/epidemic.
 Line 67: Sentence ends with "development of public health." Please be more specific, such as public health infrastructure, surveillance or interventions, etc.
 Line 75: Titter should be replaced with titer.
 Line 106: Explain CNY or write out abbreviation since it could be Chinese Yuan or Chinese New Year.
 Line 203: Sentence should read was predicted to occur around 2020 instead of is predicted.
The authors described the importance of this retrospective study and listed primary and secondary objectives of the data analysis. The background information was sufficient to understand the relevance of the study in relation to COVID-19 control measures and prevalence of Mycoplasma pneumoniae pneumonia (MPP) before, during and after the pandemic. Reference #14 is from 2012, so perhaps there is a more recent study that could be cited instead?
The study methods were described in enough detail such that someone familiar with SPSS and other standard statistical analysis tools (Pearson's chi-squared test and multi-variate logistic regression, odd ratios) could evaluate them or replicate them. As a retrospective study, there were many ways to present the data regarding demographics of children with MPP and trends in MPP based on months of admission, patient age and ward type. Complications and co-infections were presented in Tables and Figures that were color coded and easy to follow. Appropriate controls were used for the analysis.
Figures and Tables are clearly presented with a couple of exceptions. For Table 1, the manner in which the years are categorized needs additional explanation. Specifically, the Period labeled "Post-COVID period with controls" includes 2020-2021, which could be considered during the pandemic, not after the pandemic. Suggest renaming this or clarifying for the reader. The legend or notes for Table 2 needs to be corrected; specifically, the symbols used in the notes do not appear in Table 2 and extraneous letters (such as "E" on the second line of notes and the number 10 after Average cost on last line) should be removed. For Figure 5 A and B, labelling with right side of the tables as "Odds Ratio" would be helpful to the reader. Despite these suggestions, the results interpretations and conclusions are supported by the study design.
In the discussion, the authors describe the strengths of the study, but do not address any limitations or alternative explanations for the data presented. In addition, the discussion should have additional references for certain statements made as follows:
o Line 229-230: Cite the previous studies
o Line 241: Cite the US study
o Line 244: Cite the EBV reference
o Line 246: Cite the EBV reference (could be the same as line 244)
o Line 256: Reference needed for adjustment of medical insurance policies in China.

Reviewer #2 (Comments for the Author):

The article presents Mp detection before and during the COVID-19 pandemic. The prevalence of Mp has been reported from many geographic regions before and after the pandemic. The matter is of sufficient interest, the authors collected data from large sets of individuals, but the article need major detailing and significant revision.

1. The organism names need to be presented in standard format throughout the manuscript.
2. The method section in your abstract is incomplete
3. Overall, your abstract need work. It doesn't seem to show the detection rates/numbers.
4. Lane 70-76. The details of PCR/ serology etc are completely missing.
5. How are other viral/bacterial infections detected in your study population?
6. Seasonality is missing in the results section. The authors may show seasonality associated with Mp detection.
7. What are the extra pulmonary complications?
8. How the Mp cases were treated? What was the outcome? Survival vs fatality?
9. Please include Chest X ray findings of these cases if available.
10. Overall the article lacks several important details including the information of assays used. The method section is incomplete. The Introduction need improvement. Results need to be streamlined with addition of more clinical characteristics and final outcomes of these individuals. Discussion can be modified further according to your results.
11. Figures: include-statistical significance.
12. Figure3 can be modified/ present this figure as a table
13. I am not sure if the cost evaluation per CNY is standardized for global audience. In my opinion Fig 4 can be simplified.
14. Table2-please include treatment and outcome details.

Review of Manuscript # Spetrum00354-26, “Trends and Outcomes of *Mycoplasma pneumoniae* pneumonia in hospitalized children before and during the coronavirus diseases 2019 pandemic: A retrospective study in Northeast China, 2018-2023”

- General comments:
    - The manuscript is concise and organized logically so that it is easy to follow. One general comment is that *Mycoplasma pneumoniae* is not italicized throughout the document and figures/tables so this should be corrected.
    - Specific comments include the following:
        - Line 39 of the Abstract has a random “44” at the end of conclusions.
        - Lines 49-53 seems to be a run on sentence that could be reworked for ease of reading.
        - Line 53 and 55 refer to MPP as a pandemic. Please cite a reference for this or revise to say regional outbreak/epidemic.
        - Line 67: Sentence ends with “development of public health.” Please be more specific, such as public health infrastructure, surveillance or interventions, etc.
        - Line 75**:** **Titter** should be replaced with titer.
        - Line 106: Explain **CNY** or write out abbreviation since it could be Chinese Yuan or Chinese New Year.
        - Line 203: Sentence should read **was** predicted to occur around 2020 instead of **is** predicted.

The authors described the importance of this retrospective study and listed primary and secondary objectives of the data analysis. The background information was sufficient to understand the relevance of the study in relation to COVID-19 control measures and prevalence of *Mycoplasma pneumoniae* pneumonia (MPP) before, during and after the pandemic. Reference #14 is from 2012, so perhaps there is a more recent study that could be cited instead?

The study methods were described in enough detail such that someone familiar with SPSS and other standard statistical analysis tools (Pearson’s chi-squared test and multi-variate logistic regression, odd ratios) could evaluate them or replicate them.

As a retrospective study, there were many ways to present the data regarding demographics of children with MPP and trends in MPP based on months of admission, patient age and ward type. Complications and co-infections were presented in Tables and Figures that were color coded and easy to follow. Appropriate controls were used for the analysis.

Figures and Tables are clearly presented with a couple of exceptions. For Table 1, the manner in which the years are categorized needs additional explanation. Specifically, the Period labeled "Post-COVID period with controls" includes 2020-2021, which could be considered during the pandemic, not after the pandemic. Suggest renaming this or clarifying for the reader. The legend or notes for Table 2 needs to be corrected; specifically, the symbols used in the notes do not appear in Table 2 and extraneous letters (such as "E" on the second line of notes and the number 10 after Average cost on last line) should be removed. For Figure 5 A and B, labelling with right side of the tables as "**Odds Ratio**" would be helpful to the reader. Despite these suggestions, the results interpretations and conclusions are supported by the study design.

In the discussion, the authors describe the strengths of the study, but do not address any limitations or alternative explanations for the data presented. In addition, the discussion should have additional references for certain statements made as follows:

- Line 229-230: Cite the previous studies
- Line 241: Cite the US study
- Line 244: Cite the EBV reference
- Line 246: Cite the EBV reference (could be the same as line 244)
- Line 256: Reference needed for adjustment of medical insurance policies in China.

Response to the Reviewer's Comments

Thank you for your insightful and meticulous reviews of our manuscript. Your dedication to ensuring the quality and integrity of our research is truly admirable, and we are deeply appreciative of your efforts. Addressing your specific points:

1. Comment: **Mycoplasma pneumoniae is not italicized throughout the document and figures/tables so this should be corrected.**

Response: Thank you for bringing this to our attention. We have carefully checked the whole manuscript, figures and tables. All *Mycoplasma pneumoniae* have been italicized following standard scientific writing norms.

2. Comment: **Line 39 of the Abstract has a random "44" at the end of conclusions.**

Response: Thank you for bringing this to our attention. The redundant number "44" has been deleted.

3. Comment: **Lines 49-53 seems to be a run on sentence that could be reworked for ease of reading.**

Response: Thank you for bringing this to our attention. The sentence in Lines 49–53 has been revised for improved clarity and readability.

> "At the end of 2022, the Chinese government lifted these strong mitigation measures. This policy shift was soon followed by an outbreak of COVID-19 over a short period of time, concomitant with changes in the prevalence of other respiratory pathogens. Notably, MPP in children emerged as a regional epidemic in 2023." **(Lines 68-72)**

4. Comment: **Line 53 and 55 refer to MPP as a pandemic. Please cite a reference for this or revise to say regional outbreak/epidemic.**

Response: Thank you for bringing this to our attention. The relevant descriptions have been revised to "regional epidemic".

5. Comment: **Line 67: Sentence ends with "development of public health." Please be more specific, such as public health infrastructure, surveillance or interventions, etc.**

Response: Thank you for bringing this to our attention. The sentence has been revised to be more specific as suggested.

> "These data will ultimately increase public attention to MPP and contribute to the protection of children's health as well as the

development of public health infrastructure, surveillance, and interventions." **(Lines 84-86)**

6. Comment: **Line 75: Titter should be replaced with titer.**

Response: Thank you for bringing this to our attention. The spelling titter has been corrected to titer at **Line 98**.

7. Comment: **Line 106: Explain CNY or write out abbreviation since it could be Chinese Yuan or Chinese New Year.**

Response: Thank you for bringing this to our attention. CNY (Chinese Yuan) has been replaced with US dollars ($) throughout the manuscript to ensure clarity and standardization for a global readership.

8. Comment: **Line 203: Sentence should read was predicted to occur around 2020 instead of is predicted.**

Response: Thank you for bringing this to our attention. The verb tense at **Line 242** has been corrected from is to was for accuracy.

9. Comment: **Reference #14 is from 2012, so perhaps there is a more recent study that could be cited instead?**

Response: Thank you for bringing this to our attention. We have replaced the reference #14 with a more recent study published in 2025, which is now cited as reference #13.

"13 Zhang J, Wu R, Mo L, Ding J, Huang K. Trends in Mycoplasma pneumoniae infections in pediatric patient preceding, during, and following the COVID-19 pandemic: a comprehensive longitudinal analysis. Microbiol Spectr 2025;13(2):e0100124." **(Lines 391-393)**

10. Comment: **For Table 1, the manner in which the years are categorized needs additional explanation. Specifically, the Period labeled "Post-COVID period with controls" includes 2020-2021, which could be considered during the pandemic, not after the pandemic. Suggest renaming this or clarifying for the reader.**

Response: Thank you for bringing this to our attention. The grouping labels have been revised to Pre-COVID-19 period (2018–2019), During COVID-19 period with controls (2020–2022), and Post-COVID-19 period without controls (2023) for clarity.

11. Comment: **The legend or notes for Table 2 needs to be corrected; specifically, the symbols used in the notes do not appear in Table 2 and extraneous letters (such as "E" on the second line of notes and the number 10 after Average cost on last line) should be removed.**

Response: Thank you for bringing this to our attention. We have corrected the notes for Table 2 by removing symbols not appearing in the table, deleting the extraneous "E" on the second line, and removing the number "10" after "Average cost" on the last line.

12. Comment: **For Figure 5 A and B, labelling with right side of the tables as "Odds Ratio" would be helpful to the reader.**

Response: Thank you for bringing this to our attention. We have added "Odds Ratio" on the right side of the tables in Figure 5A and 5B as suggested.

13. Comment: **In the discussion, the authors describe the strengths of the study, but do not address any limitations or alternative explanations for the data presented.**

Response: Thank you for bringing this to our attention. We have added a dedicated paragraph discussing the study's limitations.

> "Our study has certain limitations. First, it was a retrospective cohort study. Second, it was a single-centre study. However, as the study was conducted at one of four National Children's Regional Medical Centres in China, the patients should accurately represent the characteristics of children with MPP in Northeast China. Finally, we focused only on hospitalized children and did not analyze children with MPP in the community." **(Lines 320-324)**

14. Comment: **Line 229-230: Cite the previous studies.**

Response: Thank you for bringing this to our attention. We have now added the citation number as reference #11.

> "11 Xing Y, Wang D, Sheng K, Xiao X, Wei H, Liu L, Zhou W, Tong X. Dynamic change of Mycoplasma pneumoniae pneumonia in hospitalized children in a general hospital: a 3-year retrospective analysis. Transl Pediatr 2020;9:522 – 31." **(Lines 385-387)**

15. Comment: **Line 241: Cite the US study.**

Response: Thank you for bringing this to our attention. We have now added the citation number as reference #20.

> "20 Diaz MH, Hersh AL, Olson J, Shah SS, Hall M, Edens C. Mycoplasma pneumoniae Infections in Hospitalized Children - United States, 2018-2024. MMWR Morb Mortal Wkly Rep. 2025 Jun 26;74(23):394-400." **(Lines 413-415)**

16. Comment: **Line 244: Cite the EBV reference.**

Response: Thank you for bringing this to our attention. We have now added the citation number as reference #21 to #23.

17. Comment: **Line 246: Cite the EBV reference (could be the same as line 244).**

Response: Thank you for bringing this to our attention. We have now added the citation number as reference #21 to #23.

18. Comment: **Line 256: Reference needed for adjustment of medical insurance policies in China.**

Response: Thank you for bringing this to our attention. We have now added the citation number as reference #30.

> "30 Yu L, Lang J. Diagnosis-related groups (DRG) pricing and payment policy in China: where are we? Hepatobiliary Surg Nutr. 2020 Dec;9(6):771-3." **(Lines 448-449)**

Response to the Reviewer's Comments

Thank you for your insightful and meticulous reviews of our manuscript. Your dedication to ensuring the quality and integrity of our research is truly admirable, and we are deeply appreciative of your efforts. Addressing your specific points:

1. Comment: **The organism names need to be presented in standard format throughout the manuscript.**

Response: Thank you for bringing this to our attention. The full manuscript has been carefully checked, and all organism names have been revised to comply with the standard academic format.

2. Comment: **The method section in your abstract is incomplete**

Response: Thank you for bringing this to our attention. The method section in the abstract has been supplemented and improved to ensure its completeness.

> "This was a retrospective cohort study of children aged 28 days to 14 years hospitalized for MPP at our institution between January 2018 and December 2023. Clinical characteristics, adverse outcomes, and associated factors were analyzed using chi-square test and multivariable logistic regression. A $p< 0.05$ was considered statistically significant." **(Lines 28-32)**

3. Comment: **Overall, your abstract need work. It doesn't seem to show the detection rates/numbers.**

Response: Thank you for bringing this to our attention. The results of abstract has been thoroughly revised, and specific detection rates and related numerical results have been added for completeness.

> "A total of 17,505 MPP cases were included, with the highest case number (4,619) in 2023. In 2023, the proportion of severe pneumonia was 44.1% and bronchoscopy usage was 33.5%, both significantly higher than in 2018–2022. Older age, admission during July–December, co-infection with Epstein-Barr virus, chlamydia, or other bacteria, and complications including pleural effusion, abnormal liver enzymes, acute respiratory distress syndrome, or atelectasis were associated with bronchoscopy. Older age, admission during October–December, co-infection with respiratory syncytial virus, adenovirus, Epstein-Barr virus, chlamydia, or other bacteria, and complications including cardiac failure, abnormal liver enzymes, myocardial damage, or anemia were associated with severe pneumonia." **(Lines 68-72)**

4. Comment: **Line 70-76. The details of PCR/ serology etc are completely missing.**

Response: Thank you for bringing this to our attention. The missing experimental details regarding PCR and serology have been supplemented.

> "Nucleic acid amplification was performed on nasopharyngeal swabs or bronchoalveolar lavage fluid using real-time polymerase chain reaction (PCR) targeting MP-specific gene fragments (e.g., P1 gene, 16S rDNA, or 16S rRNA) [9]. Alternatively, serological confirmation was based on a single serum antibody titer ≥1:160 (detected by particle agglutination assay), or seroconversion and/or a ≥4-fold antibody titer increase in acute and convalescent paired serum samples collected at an interval of ≥2 weeks [9]." **(Lines 95-101)**

5. Comment: **How are other viral/bacterial infections detected in your study population?**

Response: Thank you for bringing this to our attention. The detection methods for other viral and bacterial infections in the study population have been supplemented and described in detail in the revised manuscript.

> "Co-infection with other common respiratory pathogens, including respiratory syncytial virus (RSV), Epstein-Barr virus (EBV), adenovirus (Adv), influenza virus, Chlamydia, COVID-19, and other bacteria, was detected using commercial multiplex PCR panels on nasopharyngeal swab combined with routine bacterial culture of respiratory specimens." **(Lines 123-127)**

6. Comment: **Seasonality is missing in the results section. The authors may show seasonality associated with Mp detection.**

Response: Thank you for bringing this to our attention. To make the seasonality results more visible and easier to locate, we have now highlighted the relevant sentences in red in the revised Results section.

> "In 2023, hospital admissions were highest from October to December (n=2318), increasing gradually throughout the year, and peaking in November. From 2018 to 2023, hospital admissions increased between September and November, although to a much lesser extent than in 2023, showing a downward trend after November (Table 1, Fig. 2). In terms of ward type, the number of patients admitted to the PICU was the highest in 2019 (Table 2, Fig. 3b)." **(Lines 177-182)**

7. Comment: **What are the extra pulmonary complications?**

Response: Thank you for bringing this to our attention. To make the extrapulmonary complications more visible and easier to locate, we have now highlighted the relevant sentences in red in the revised Results section.

> "extrapulmonary complications (including myocardial damage, cardiac failure, abnormal liver enzyme indicators, sepsis, skin involvement, arterial embolism, laryngitis, and encephalitis)" **(Lines 116-118)**

8. Comment: **How the Mp cases were treated? What was the outcome? Survival vs fatality?**

Response: Thank you for bringing this to our attention. We have supplemented detailed descriptions regarding the treatment regimens, clinical outcomes of MPP in the revised manuscript.

> "M. pneumoniae infections exhibit a certain degree of self-limitation, with most cases being mild and associated with a favorable prognosis [25]. However, some patients may progress to pneumonia or develop severe disease accompanied by pulmonary and/or extrapulmonary complications, and in severe cases, death may occur [25]. In our study, 20 children hospitalized with MPP died between 2018 and 2023. Macrolide antibiotics are the preferred medication for MPP in children [25]. Owing to their extensive use, MRMP has emerged and spread globally for over 20 years, with prevalence rates exceeding 90% in China [26-28]. Studies suggest that tetracyclines and fluoroquinolones may be used as second-line therapies for the treatment of pediatric MRMP [29]." **(Lines 292-300)**

9. Comment: **Please include Chest X ray findings of these cases if available.**

Response: Thank you for the valuable suggestion. Unfortunately, as this is a retrospective cohort study based on routine clinical electronic medical records, standardized chest X-ray imaging data were not systematically archived for all enrolled cases. Therefore, we are unable to supplement the chest X-ray findings in this manuscript.

10. Comment: **Overall the article lacks several important details including the information of assays used. The method section is incomplete. The Introduction need improvement. Results need to be streamlined with addition of more clinical characteristics and final outcomes of these individuals. Discussion can be modified further according to your results.**

Response: Thank you very much for your comprehensive and constructive comments. We have carefully revised the manuscript accordingly: the missing experimental assay details have been supplemented to complete the Methods section; the Introduction has been thoroughly improved; the Results have been streamlined, with more clinical

characteristics and patient final outcomes added; and the Discussion has also been further modified and refined based on our present findings. All changes are highlighted in the revised manuscript.

11. Comment: **Figures: include-statistical significance.**

Response: We have regenerated all figures as suggested. Where applicable, statistical significance has now been added to the figures to indicate significant differences.

12. Comment: **Figure 3 can be modified/ present this figure as a table**

Response: Thank you for bringing this to our attention. In the revised manuscript, Figure 3 has been removed, and its content is now presented in Table 2 for better clarity and data organization.

13. Comment: **I am not sure if the cost evaluation per CNY is standardized for global audience. In my opinion Fig 4 can be simplified.**

Response: Thank you for bringing this to our attention. CNY (Chinese Yuan) has been replaced with US dollars ($) throughout the manuscript to ensure clarity and standardization for a global readership. We have simplified Figure 4 as suggested.

14. Comment: **Table 2-please include treatment and outcome details.**

Response: Thank you for bringing this to our attention. We have supplemented the detailed information on treatment regimens and clinical outcomes in Table 2 as suggested.

Re: Spectrum00354-26R1 (**Trends and outcomes of *Mycoplasma pneumoniae* pneumonia in hospitalized children before, during and after the coronavirus disease 2019 pandemic: A retrospective study in Northeast China, 2018-2023**)

Dear Prof. Kai You:

Thank you for the privilege of reviewing your work. This work has been much improved by the changes. The reviewers pointed out minor additional comments. Below you will find my comments, instructions from the Spectrum editorial office, and the reviewer comments.

Please return the manuscript within 60 days; if you cannot complete the modification within this time period, please contact me. If you do not wish to modify the manuscript and prefer to submit it to another journal, notify me immediately so that the manuscript may be formally withdrawn from consideration by Spectrum.

Revision Guidelines
To submit your modified manuscript, log into the submission site at https://spectrum.msubmit.net/cgi-bin/main.plex. Go to Author Tasks and click the appropriate manuscript title to begin. The information you entered when you first submitted the paper will be displayed; update this as necessary. Note the following requirements:

• Upload point-by-point responses to the issues raised by the reviewers in a file named "Response to Reviewers," NOT in your cover letter.
• Upload a compare copy of the manuscript (without figures) as a "Marked-Up Manuscript" file.
• Upload a clean .DOC/.DOCX version of the revised manuscript and remove the previous version.
• Each figure must be uploaded as a separate, editable, high-resolution file (TIFF or EPS preferred), and any multipanel figures must be assembled into one file.
• Any supplemental material intended for posting by ASM should be uploaded with their legends separate from the main manuscript. You can combine all supplemental material into one file (preferred) or split it into a maximum of 10 files with all associated legends included.

For complete guidelines on revision requirements, see our Submission and Review Process webpage. Submission of a paper that does not conform to guidelines may delay acceptance of your manuscript.

Data availability: ASM policy requires that data be available to the public upon online posting of the article, so please verify all links to sequence records, if present, and make sure that each number retrieves the full record of the data. If a new accession number is not linked or a link is broken, provide Spectrum production staff with the correct URL for the record. If the accession numbers for new data are not publicly accessible before the expected online posting of the article, publication may be delayed; please contact production staff (Spectrum@asmusa.org) immediately with the expected release date.

Publication Fees: For information on publication fees and which article types are subject to charges, visit our website. If your manuscript is accepted for publication and any fees apply, you will be contacted separately about payment during the production process; please follow the instructions in that e-mail. Arrangements for payment must be made before your article is published.

ASM Membership: Corresponding authors may join or renew ASM membership to obtain discounts on publication fees. Need to upgrade your membership level? Please contact Customer Service at Service@asmusa.org.

The ASM Journals program strives for constant improvement in our submission and publication process. Please tell us how we can improve your experience by taking this quick Author Survey.

Thank you for submitting your paper to Spectrum.

Sincerely,
Cecilia Thompson
Editor
Microbiology Spectrum

Reviewer #1 (Comments for the Author):

I reviewed all the changes requested, and all changes have been addressed by the authors. Based on the changes, I recommend acceptance.

Reviewer #2 (Comments for the Author):

Thank you for responding to my queries. The article's quality improved slightly but not significantly. Some suggestions are attached

- Abstract Lane 23, 26 Mycoplasma pneumoniae (MPP) is repeated.
- In Results, treatment part is mentioned very briefly without many details.
- Lane 289-294-discuss your experience. How were your patients treated and responded? This would make the article very interesting.

Response to the Reviewer's Comments

Thank you for your insightful and meticulous reviews of our manuscript. Your dedication to ensuring the quality and integrity of our research is truly admirable, and we are deeply appreciative of your efforts. Addressing your specific points:

1. Comment: **Abstract Lane 23, 26 Mycoplasma pneumoniae (MPP) is repeated.**

Response: Thank you for bringing this to our attention. We have removed the duplicate "Mycoplasma pneumoniae (MPP)" in the abstract.

2. Comment: **In Results, treatment part is mentioned very briefly without many details.**

Response: Thank you for bringing this to our attention. We have revised the treatment part in the Results section accordingly.

> "In addition to pharmacotherapy, including macrolides, tetracyclines, glucocorticoids, among others, various procedural interventions and respiratory support measures were applied in a subset of patients. Among procedural interventions, the utilization rates of bronchoscopy (33.5%) and thoracocentesis (1.1%) were significantly higher in 2023 than in 2018–2022. Regarding respiratory support, the proportions of patients receiving oxygen therapy (8.6%) and non-invasive mechanical ventilation (1.3%) were highest in 2020 among the six study years." **(Lines 190-196)**

3. Comment: **Lane 289-294-discuss your experience. How were your patients treated and responded? This would make the article very interesting.**

Response: Thank you for bringing this to our attention. We have expanded the Discussion to share our treatment experience. In our center, macrolides were used as first-line therapy. For patients failing to respond after 72 hours, we considered MRMP and shifted to second-line agents, including tetracyclines or fluoroquinolones. Systemic glucocorticoids (methylprednisolone, 2 mg/kg/day, escalated to 4–6 mg/kg/day for severe cases with extensive consolidation or markedly elevated inflammatory markers) were added for severe or critical cases. Bronchoscopy was performed early in children with suspected mucus plug obstruction or plastic bronchitis, and intravenous immunoglobulin was reserved for those with severe extrapulmonary complications. For patients with hypoxemia, we applied a stepwise respiratory support strategy—oxygen therapy, non-invasive ventilation, and invasive mechanical ventilation—according to the severity of respiratory failure. Additional procedures, including thoracocentesis, closed drainage, plasma exchange, and lumbar puncture, were performed as

clinically indicated. Through this comprehensive approach, most patients achieved favorable responses, and overall mortality remained low.

> "In our center, macrolides remained the first-line therapy for initial MPP cases. For patients who failed to show clinical improvement after 72 hours of macrolide treatment, we considered the possibility of MRMP and shifted to second-line antibiotics, such as tetracyclines or fluoroquinolones. Systemic glucocorticoids. Bronchoscopy was performed early in severely ill children suspected of having mucus plug obstruction or plastic bronchitis. Intravenous immunoglobulin was reserved for cases with severe extrapulmonary complications such as encephalitis, severe mucocutaneous lesions, or hematological manifestations." **(Lines 303-311)**

Re: Spectrum00354-26R2 (**Trends and outcomes of *Mycoplasma pneumoniae* pneumonia in hospitalized children before, during and after the coronavirus disease 2019 pandemic: A retrospective study in Northeast China, 2018-2023**)

Dear Prof. Kai You:

Your manuscript has been accepted, and I am forwarding it to the ASM production staff for publication. Your paper will first be checked to make sure all elements meet the technical requirements. ASM staff will contact you if anything needs to be revised before copyediting and production can begin. Otherwise, you will be notified when your proofs are ready to be viewed.

Data Availability: ASM policy requires that data be available to the public upon online posting of the article, so please verify all links to sequence records, if present, and make sure that each number retrieves the full record of the data. If a new accession number is not linked or a link is broken, provide production staff with the correct URL for the record. If the accession numbers for new data are not publicly accessible before the expected online posting of the article, publication may be delayed; please contact ASM production staff immediately with the expected release date.

Publication Fees: For information on publication fees and which article types have charges, please visit ourwebsite. We have partnered with Copyright Clearance Center (CCC) to collect author charges. If fees apply to your paper, you will receive a message from no-reply@copyright.com with further instructions. For questions related to paying charges through RightsLink, please contact CCC at ASM_Support@copyright.com or toll free at +1-877-622-5543. CCC makes every attempt to respond to all emails within 24 hours.

ASM Membership: Corresponding authors may join or renew ASM membership to obtain discounts on publication fees. Need to upgrade your membership level? Please contact Customer Service at Service@asmusa.org.

Embargo Policy: A press release may be issued as soon as the manuscript is posted on theSpectrum Latest Articles webpage. The corresponding author will receive an email with the subject line "ASM Journals Author Services Notification" when the article is available online.

Cover Image Submissions: If you would like to submit a potential Cover Image, please email a file and a short legend to spectrum@asmusa.org. Please note that we can only consider images that (i) the authors created or own and (ii) have not been previously published. By submitting, you agree that the image can be used under the same terms as the published article. Image File requirements: TIF/EPS, 7.5 inches wide by 8.25 inches tall (at least 2,250 pixels wide by 2,475 pixels tall), minimum 300 dpi resolution (600 dpi preferred), RGB, and no figure elements, e.g., arrows or panel labels. The legend should be a short description of the image, 1-2 sentences recommended. Please download and use this interactive template in Adobe the https://journals.asm.org/pb-assets/html/asm-cover-sizing-tool.html to ensure that your proposed cover image meets our size requirements (https://journals.asm.org/pb-assets/pdf-text-excel-files/ASM-Interactive-Sizing-Cover-Template-1715689791.pdf).

The ASM Journals program strives for constant improvement in our submission and publication process. Please tell us how we can improve your experience by taking this quick Author Survey.

Thank you for submitting your paper to Spectrum.

Sincerely,
Cecilia Thompson
Editor
Microbiology Spectrum
