# Supplementary material for: Trends and outcomes of Mycoplasma pneumoniae pneumonia in hospitalized children before, during, and after the coronavirus disease 2019 pandemic: a retrospective study in Northeast China, 2018–2023
Source: Microbiol Spectr. 2026 Aug 14;14(9):e00354-26. doi: 10.1128/spectrum.00354-26 (PMC13532259; doi:10.1128/spectrum.00354-26)
Supplement: Supplementary file 2 [file spectrum.00354-26-s0001.docx]

**Supplementary Table 1.** [Univariate](https://zhuanlan.zhihu.com/p/344682163) logistic regression analysis of factors associated with respiratory failure, duration of hospital stay, and total treatment cost

| Variable | Respiratory failure | | Duration of hospitalization | | Total cost | |
| --- | --- | --- | --- | --- | --- | --- |
|  | *p* value | OR (95% CI) | *p* value | OR (95% CI) | *p* value | OR (95% CI) |
| Age, years |  |  |  |  |  |  |
| 0–1 |  | 1.00 (Reference) |  | 1.00 (Reference) |  | 1.00 (Reference) |
| 1–3 | <.001 | 0.56 (0.40 ~ 0.78) | 0.163 | 0.92 (0.83 ~ 1.03) | 0.029 | 0.88 (0.79 ~ 0.99) |
| 3–6 | <.001 | 0.45 (0.30 ~ 0.68) | 0.703 | 0.98 (0.87 ~ 1.10) | 0.032 | 0.88 (0.78 ~ 0.99) |
| >6 | 0.007 | 0.59 (0.40 ~ 0.87) | 0.105 | 0.91 (0.80 ~ 1.02) | 0.002 | 0.82 (0.73 ~ 0.93) |
| Quarter |  |  |  |  |  |  |
| January to March |  | 1.00 (Reference) |  | 1.00 (Reference) |  | 1.00 (Reference) |
| April to June | 0.076 | 0.72 (0.50 ~ 1.04) | 0.062 | 0.91 (0.82 ~ 1.00) | <.001 | 0.67 (0.60 ~ 0.74) |
| July to September | <.001 | 0.54 (0.38 ~ 0.77) | 0.017 | 1.11 (1.02 ~ 1.22) | 0.006 | 0.88 (0.81 ~ 0.96) |
| October to December | 0.114 | 0.78 (0.58 ~ 1.06) | <.001 | 0.78 (0.72 ~ 0.85) | <.001 | 0.70 (0.64 ~ 0.77) |
| Co-infected with RSV |  |  |  |  |  |  |
| No |  | 1.00 (Reference) |  | 1.00 (Reference) |  | 1.00 (Reference) |
| Yes | 0.034 | 1.68 (1.04 ~ 2.73) | 0.077 | 1.16 (0.98 ~ 1.37) | 0.013 | 1.24 (1.05 ~ 1.47) |
| Co-infected with EB |  |  |  |  |  |  |
| No |  | 1.00 (Reference) |  | 1.00 (Reference) |  | 1.00 (Reference) |
| Yes | 0.030 | 0.66 (0.46 ~ 0.96) | <.001 | 1.16 (1.07 ~ 1.26) | <.001 | 1.18 (1.09 ~ 1.29) |
| Co-infected with Adv |  |  |  |  |  |  |
| No |  | 1.00 (Reference) |  | 1.00 (Reference) |  | 1.00 (Reference) |
| Yes | 0.001 | 2.19 (1.37 ~ 3.49) | <.001 | 1.46 (1.22 ~ 1.73) | <.001 | 1.81 (1.51 ~ 2.16) |
| Co-infected with influenza virus |  |  |  |  |  |  |
| No |  | 1.00 (Reference) |  | 1.00 (Reference) |  | 1.00 (Reference) |
| Yes | 0.323 | 1.42 (0.71 ~ 2.85) | 0.521 | 1.08 (0.86 ~ 1.36) | <.001 | 1.94 (1.52 ~ 2.49) |
| Co-infected with other bacteria |  |  |  |  |  |  |
| No |  | 1.00 (Reference) |  | 1.00 (Reference) |  | 1.00 (Reference) |
| Yes | 0.036 | 1.86 (1.04 ~ 3.33) | <.001 | 1.93 (1.53 ~ 2.43) | <.001 | 2.03 (1.59 ~ 2.59) |
| Anemia |  |  |  |  |  |  |
| No |  | 1.00 (Reference) |  | 1.00 (Reference) |  | 1.00 (Reference) |
| Yes | <.001 | 2.66 (1.75 ~ 4.03) | <.001 | 1.65 (1.37 ~ 1.99) | <.001 | 1.88 (1.55 ~ 2.28) |
| Pleural effusion |  |  |  |  |  |  |
| No |  | 1.00 (Reference) |  | 1.00 (Reference) |  | 1.00 (Reference) |
| Yes | 0.002 | 1.79 (1.23 ~ 2.60) | <.001 | 2.07 (1.83 ~ 2.34) | <.001 | 3.23 (2.83 ~ 3.69) |
| Atelectasis |  |  |  |  |  |  |
| No |  | 1.00 (Reference) |  |  |  | 1.00 (Reference) |
| Yes | 0.004 | 3.43 (1.48 ~ 7.96) |  |  | <.001 | 3.37 (2.15 ~ 5.28) |
| Pneumothorax |  |  |  |  |  |  |
| No |  | 1.00 (Reference) |  | 1.00 (Reference) |  | 1.00 (Reference) |
| Yes | <.001 | 10.86 (4.68 ~ 25.21) | 0.025 | 2.24 (1.11 ~ 4.52) | <.001 | 9.96 (3.04 ~ 32.63) |
| Myocardial damage |  |  |  |  |  |  |
| No |  | 1.00 (Reference) |  | 1.00 (Reference) |  | 1.00 (Reference) |
| Yes | <.001 | 7.39 (4.38 ~ 12.47) | 0.065 | 1.40 (0.98 ~ 2.00) | <.001 | 2.59 (1.74 ~ 3.86) |
| Cardiac failure |  |  |  |  |  |  |
| No |  | 1.00 (Reference) |  | 1.00 (Reference) |  | 1.00 (Reference) |
| Yes | <.001 | 40.93 (17.81 ~ 94.06) | 0.077 | 2.13 (0.92 ~ 4.95) | 0.002 | 6.72 (2.00 ~ 22.61) |
| Abnormal liver enzyme indicators |  |  |  |  |  |  |
| No |  | 1.00 (Reference) |  | 1.00 (Reference) |  | 1.00 (Reference) |
| Yes | <.001 | 2.10 (1.38 ~ 3.21) | <.001 | 1.68 (1.40 ~ 2.01) | <.001 | 1.68 (1.40 ~ 2.01) |

Adv, adenovirus; CI, confidence interval; EBV, Epstein-Barr virus; OR, odds ratio; RSV, respiratory syncytial virus.

**Supplementary Table 2.** Trends in the distribution of costs according to ward type

|  | Total | PICU | Ward |
| --- | --- | --- | --- |
|  | n=17505 | n=1466 | n=16039 |
| Total cost (×10⁶ $) | 28.60 | 5.24 | 23.36 |
| Medical service fee (×10⁶ $) | 6.99 | 1.58 | 5.40 |
| Medication (×10⁶ $) | 6.11 | 1.14 | 4.97 |
| Diagnostic test (×10⁶ $) | 0.45 | 0.07 | 0.37 |
| Laboratory test (×10⁶ $) | 4.64 | 0.78 | 3.86 |
| Imaging test (×10⁶ $) | 0.62 | 0.11 | 0.52 |
| Medical material fee (×10⁶ $) | 1.73 | 0.57 | 1.16 |
| Others (×10⁶ $) | 8.11 | 0.99 | 7.12 |

PICU, pediatric intensive care unit.

**Supplementary Table 3.** Trends in the distribution of costs according to year

|  | 2018 | 2019 | 2020 | 2021 | 2022 | 2023 |
| --- | --- | --- | --- | --- | --- | --- |
|  | n=3518 | n=4006 | n=1674 | n=2132 | n=1556 | n=4619 |
| Total cost (×10⁶ $) | 5.89 | 7.34 | 3.13 | 2.96 | 2.51 | 6.77 |
| Medical service fee (×10⁶ $) | 1.41 | 2.13 | 0.66 | 0.59 | 0.60 | 2.10 |
| Medication (×10⁶ $) | 1.47 | 1.76 | 0.63 | 0.59 | 0.48 | 1.56 |
| Diagnostic test (×10⁶ $) | 0.12 | 0.15 | 0.03 | 0.03 | 0.03 | 0.14 |
| Laboratory test (×10⁶ $) | 0.94 | 1.17 | 0.53 | 0.50 | 0.38 | 1.08 |
| Imaging test (×10⁶ $) | 0.12 | 0.15 | 0.06 | 0.06 | 0.08 | 0.14 |
| Medical material fee (×10⁶ $) | 0.35 | 0.44 | 0.19 | 0.18 | 0.15 | 0.41 |
| Others (×10⁶ $) | 1.47 | 1.54 | 1.03 | 1.01 | 0.80 | 1.35 |
| Total cost (×10⁶ $) | 1.41 | 2.13 | 0.66 | 0.59 | 0.60 | 2.10 |
